# Abiotrophia defectiva Endocarditis: A Case Diagnosed Incidentally Due to Multi-organ Embolism

**DOI:** 10.7759/cureus.63146

**Published:** 2024-06-25

**Authors:** Fumitaka Suzuki, Masahiro Tsutsui, Hideki Isa, Shingo Kunioka, Hiroyuki Kamiya

**Affiliations:** 1 Cardiac Surgery, Asahikawa Medical University, Asahikawa, JPN

**Keywords:** aortic valve plasty, mitral valve replacement, multi organ embolism, infectious endocarditis, abiotrophia defectiva

## Abstract

*Abiotrophia defectiva*, often referred to as nutritionally variant streptococci, is generally a benign part of human microflora, primarily found in the oral cavity, digestive tract, and genitourinary system. However, it can have a significant role in infectious endocarditis (IE). We discuss a case involving a 53-year-old male who displayed serious signs indicative of IE. The individual, who had a history of IgA nephropathy, underwent successful surgical and antibiotic intervention. Given the challenge in treating *A. defectiva* due to its high antibiotic resistance and the tendency for embolic events and treatment failure, a multidimensional approach involving surgical intervention and specific antibiotic therapy resulted in a successful outcome. This case underlines the need for early identification, immediate treatment, and additional research to understand better and manage *A. defectiva* endocarditis.

## Introduction

*Abiotrophia defectiva*, often referred to as nutritionally variant streptococci, is typically a benign component of human microflora. It resides primarily in regions such as the oral cavity, digestive tract, and the genitourinary system [1]. Despite its seemingly harmless nature, it holds clinical significance due to its connection with conditions like septicemia, conjunctivitis, and otitis media and severe complications like pancreatic abscesses. One of its key roles is in the development of infective endocarditis (IE), accounting for 5-6% of IE instances linked to viridans streptococci [2]. Moreover, IE due to *A. defectiva* has been known to cause massive embolism; therefore, early diagnosis and prompt therapeutic intervention are of paramount importance [3,4]. Here, we report a case of incidentally diagnosed *A. defectiva* IE presenting multiple cerebral infarctions and splenic embolism.

## Case presentation

**Figure 1:**
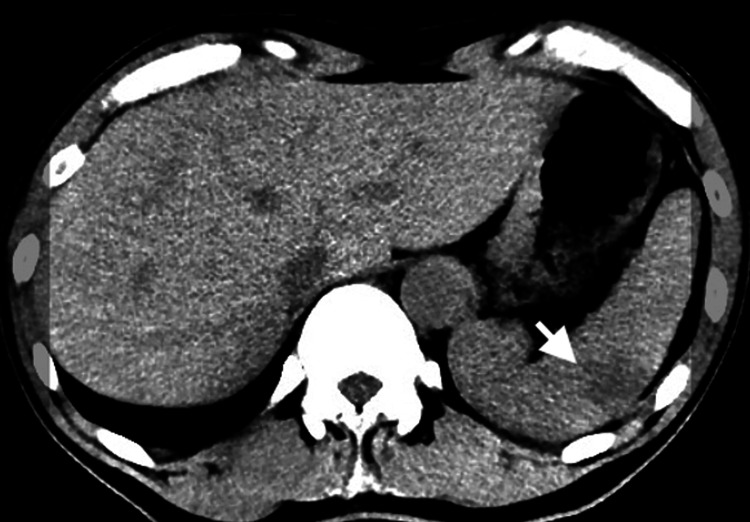
Splenic infarction on abdomen CT Complications of *Abiotrophia defectiva* infective endocarditis.

**Figure 2:**
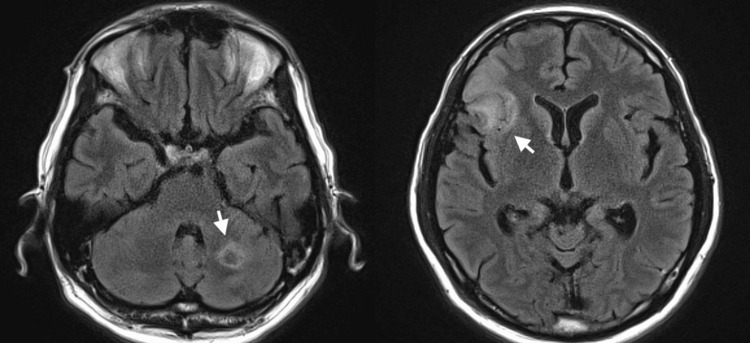
Multiple cerebral infarcts on brain MRI Complications of *Abiotrophia defectiva* infective endocarditis.

**Figure 3:**
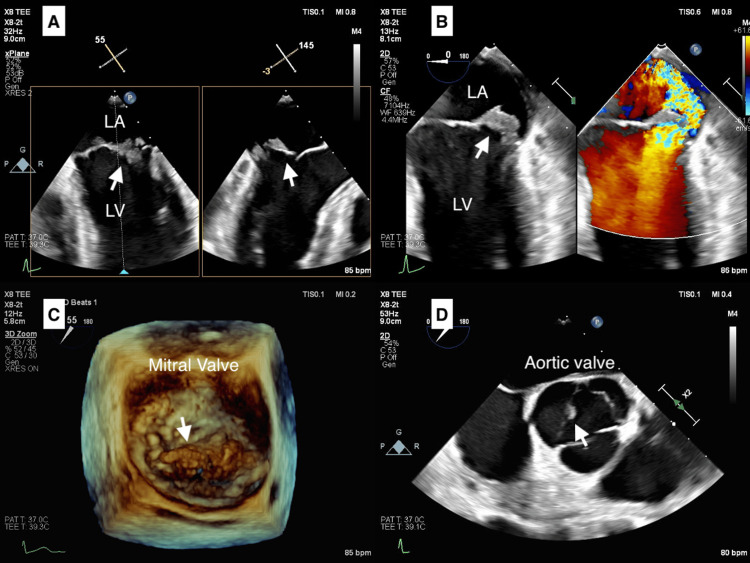
Transesophageal echocardiogram (TEE) (A) TEE shows vegetation on the mitral valve. (B) Severe mitral regurgitation. (C) 3D view of vegetation on the mitral valve. (D) Vegetation on the non-coronary cusp of the aortic valve.

A 53-year-old male, managed as an outpatient for IgA nephropathy, reported ongoing fatigue, night sweats, sporadic fevers, and a substantial weight loss of 15 kg over half a year. While running a computed tomography scan under the suspicion of cancer, an unexpected splenic infarct was discovered (Figure 1), and multiple cerebral infarctions were detected (Figure 2) with a magnetic resonance imaging scan of his head. Due to the suspicion of IE, an initial antibiotic regimen comprising vancomycin and ceftriaxone was administered, and the patient was referred to our medical care. On admission to our hospital, blood tests showed an elevated inflammatory response with elevated white blood cell counts and elevated C-reactive protein. The patient had no heart failure symptoms and an NT-pro BNP of 638 pg/mL. Transthoracic echocardiography showed severe mitral regurgitation and findings of suspected vegetation on the mitral and aortic valves (Figure 3). On the fourth day following admission, *A. defectiva* was identified from blood cultures taken by the previous physician, prompting a switch in the antibiotic regimen to ampicillin and gentamicin. Six days into his admission, the patient was subjected to mitral valve replacement (MVR) and aortic valve plasty. MVR was performed using a 25 mm mechanical valve (St. Jude Medical, Inc., St. Paul, MN), and the aortic valve was formed with a bovine pericardial patch as a small hole had developed at the site of the resected vegetation of the noncoronary cusp. No bacteria were detected in the mitral valve vegetation, but *A. defectiva* was detected in the aortic valve vegetation. He was then discharged home with an uneventful postoperative course after six weeks of specialized antibiotic therapy. Yearly echocardiographic examinations are planned for the future.

## Discussion

*A. defectiva*, a regular inhabitant of the flora in oral, genitourinary, and intestinal tracts, is detected in nearly 11.8% of healthy adult oral cavities [5]. Its contribution to IE is substantial, accounting for roughly 5-6% of IE cases that are tied to viridans streptococci and less than 1% of all cases of endocarditis reported in Europe and North America [6]. This bacterium produces exopolysaccharides, which exhibit a strong affinity for the endocardium [7], adding to its pathogenicity. Managing IE caused by *A. defectiva* poses a significant challenge due to high relapse rates and the occurrence of embolic events [8,9]. Despite suitable antibiotic treatment, about 41% [10] of cases are reported to experience treatment failure. IE attributed to *A. defectiva* typically exhibits a protracted course of progression. Despite the organism’s susceptibility to antimicrobial agents, a substantial proportion, nearly 50%, of cases necessitate surgical intervention for effective management [11]. In this specific case, multiple embolic events transpired in the brain and spleen. The recommended treatment protocol includes a combination of either penicillin or ampicillin and gentamicin, to be administered for four to six weeks. About 50% of *A. defectiva* shows resistance to beta-lactam antibiotics, and a striking 93% is resistant to macrolide antibiotics. Despite these resistance factors, the combined use of penicillin and gentamicin is deemed more effective than the sole use of penicillin [12,13]. Our case demonstrates the crucial need for early identification and prompt intervention when dealing with *A. defectiva* endocarditis. Even though the patient encountered serious embolic events, a positive clinical trajectory was achieved through a double valve replacement operation and a suitable course of antibiotics. This case underscores the importance of adopting an all-inclusive approach to manage *A. defectiva* endocarditis, which calls for appropriate surgical procedures and specifically designed antibiotic treatment regimens. Further exploration into this elusive bacterium is necessitated to deepen our understanding of its pathogenesis, optimal diagnostic approaches, and the most effective treatment plans.

## Conclusions

In conclusion, this case underscores the aggressive nature of *A. defectiva* in the manifestation of IE. The clinical presentation of the condition is frequently malignant, characterized by multi-organ embolism that necessitates early and decisive medical intervention. A multi-pronged therapeutic approach, incorporating immediate diagnosis, vigilant antibiotic management, and, where necessary, surgical intervention, is imperative to mitigate the severe complications associated with this pathogen. Health practitioners should maintain a high index of suspicion for *A. defectiva* IE, ensuring that patients receive timely and appropriate care to optimize clinical outcomes.
